# A novel YOLOv3-arch model for identifying cholelithiasis and classifying gallstones on CT images

**DOI:** 10.1371/journal.pone.0217647

**Published:** 2019-06-18

**Authors:** Shanchen Pang, Tong Ding, Sibo Qiao, Fan Meng, Shuo Wang, Pibao Li, Xun Wang

**Affiliations:** 1 College of Computer and Communication Engineering, China University of Petroleum, Qingdao, Shandong, China; 2 Shandong Provincial Third Hospital, Jinan, Shandong, China; Polytechnical Universidad de Madrid, SPAIN

## Abstract

Locating diseases precisely from medical images, like ultrasonic and CT images, have been one of the most challenging problems in medical image analysis. In recent years, the vigorous development of deep learning models have greatly improved the accuracy in disease location on medical images. However, there are few artificial intelligent methods for identifying cholelithiasis and classifying gallstones on CT images, since no open source CT images dataset of cholelithiasis and gallstones is available for training the models and verifying their performance. In this paper, we build up the first medical image dataset of cholelithiasis by collecting 223846 CT images with gallstone of 1369 patients. With these CT images, a neural network is trained to “pick up” CT images of high quality as training set, and then a novel Yolo neural network, named Yolov3-arch neural network, is proposed to identify cholelithiasis and classify gallstones on CT images. Identification and classification accuracies are obtained by 10-fold cross-validations. It is obtained that our Yolov3-arch model is with average accuracy 92.7% in identifying granular gallstones and average accuracy 80.3% in identifying muddy gallstones. This achieves 3.5% and 8% improvements in identifying granular and muddy gallstones to general Yolo v3 model, respectively. Also, the average cholelithiasis identifying accuracy is improved to 86.50% from 80.75%. Meanwhile, our method can reduce the misdiagnosis rate of negative samples by the object detection model.

## Introduction

Cholelithiasis accompanying with gallstones is one of the most common and costly diseases in population, which is with an estimated prevalence of 10-20%. Statistically, symptomatic disease is responsible for 1.4 million visits and 750 000 cholecystectomies per year in the United States. More than 75% cholelithiasis patients belong to cholesterol or cholesterol-predominant type [1]. There are two fundamental features of cholelithiasis, composition and location. By chemical composition, gallstones can be separated into three classes: cholesterol-like gallstones, bile pigmented gallstones, and mixed gallstones. Considering the locations, there are three kinds of gallstones: gallbladder stones, intrahepatic bile duct stones, common hepatic bile duct stones. Identifying cholelithiasis and classifying the type of gallstones precisely on CT images is one of the most challenging problems in medical image analysis.

Recently, with a widely open data movement, the vigorous development of deep learning methods have greatly improved ability of computer-assisted diagnosis method [2]. There are three major tasks in medical image analysis, which are image classification, detection and segmentation. Image classification focuses on dividing images into a single category, which corresponds to the most prominent object on the image. Since many images in the real world may contain more than one objects, it is rough and inaccurate to assign a single label to the image using the image classification model. Disease detection model can recognize multiple objects from medical images and locate different objects (give the boundary box) [3], which is useful in many scenarios, such as driverless and security systems.

Current mainstream object detection algorithms are mainly with deep learning models, belonging to one of the following two categories.

1. Two-stage detection algorithm. It firstly generates candidate region (region proposals), and then the candidate area classification can be updated by refinement, see e.g., R-CNN [4], Fast R-CNN [5], and Faster-R-CNN [6] etc.
2. One-stage detection algorithm. It directly generates the category probability and position coordinate values of objects, see e.g. Yolo [7] and SSD [8].

It is known that two-stage detection algorithms perform well in identifying accuracy, while one-stage detection algorithms is significant in less time cost. Since gallbladder diseases including cholecystitis and gallstones can be diagnosed by using ultra-sonographic examinations, there are some works on segmenting the gallbladder and gallstones in ultrasound images [9].

There are some other state-of-art objects detection models. Cascade R-CNN [10], which consists of a sequence of detectors trained with increasing IoU thresholds, to be sequentially more selective against close false positives. The detectors are trained stage by stage, leveraging the observation that the output of a detector is a good distribution for training the next higher quality detector. The resampling of progressively improved hypotheses guarantees that all detectors have a positive set of examples of equivalent size, reducing the overfitting problem. Relation networks for object detection [11] processes a set of objects simultaneously through interaction between their appearance feature and geometry, thus allowing modeling of their relations. Single-Shot refinement neural network [12] takes the refined anchors as the input from the former to further improve the regression accuracy and predict multi-class label. Meanwhile, we design a transfer connection block to transfer the features in the anchor refinement module to predict locations, sizes and class labels of objects in the object detection module. Since small and large objects are difficult to recognize at smaller and larger scales respectively, Bharat present a novel training scheme called Scale Normalization for Image Pyramids (SNIP) [13] which selectively back-propagates the gradients of object instances of different sizes as a function of the image scale.

Deep learning methods have been found useful in clinical application, and have some successful achievements [14–18]. However, few deep learning methods are designed for identifying cholelithiasis and classifying gallstones on CT images, since no open source CT images dataset of cholelithiasis and gallstones is available for training the models and verifying their performance. This arouses the application of deep learning methods for identifying cholelithiasis and classifying gallstones on CT Images. It is known that the training set of cholecystitis and gallstone is fundamental and crucial for using deep learning models. But, till now, there is no open source dataset of cholecystitis and gallstone with labels.

In this paper, we build up the first dataset of cholecystitis and gallstones by collecting 223846 CT images of 1369 cholecystitis patients with gallstones. The patients are from The Third Hospital of Shandong Province in China during 2011 to 2017, with private information being removed. The labels of cholecystitis and gallstones are determined by imaging doctors, and all patients have chosen surgical intervention to take away the gallstones.

Initially, we manually pick out 1065 high quality CT images with liver and gall (small part of the original data set and easily pick out by manual works), and train a neural network to “pick out” CT images containing liver and gall of high quality. This processed data set contains 5986 CT images and few of them are misjudged. After that, a novel Yolo framework, called Yolov3-arch model, is proposed for identifying cholelithiasis and classifying gallstones on CT images. Identification and classification accuracies are obtained by 10-fold cross-validations. The main performance indicator of the object detection model is mean average precision (mAP). It is obtained that our Yolov3-arch model is with average accuracy 92.7% in identifying granular gallstones and average accuracy 80.3% in identifying muddy gallstones. This achieves 3.5% and 8% improvements in identifying granular and muddy gallstones to general Yolo v3 model, respectively. Also, the average cholelithiasis identifying accuracy is improved to 86.50% from 80.75%. Meanwhile, our method can reduces the misdiagnosis rate of negative samples by the object detection model. For negative samples (there is no gallstone in CT image), we acquire 96 CT images that are misjudged by general Yolo-V3 network, while use our model all 96 CT images were right judged.

## Materials and methods

This research was allowed by Shandong Provincial Third Hospital and was carried out with the College of Computer and Communication Engineering, China University of Petroleum (East China), Shandong, China. The ethics committee has approved research and the college has reached a research agreement with the Shandong Provincial Third Hospital. All patients are anonymous and have no personal information.

### Related model of objects detection

**Fig 1:**
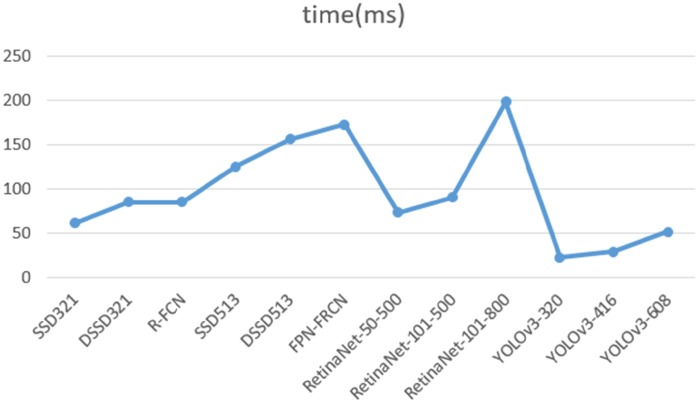
Performance of Yolo-v3 and other deep models on the COCO dataset [19].

**Fig 2:**
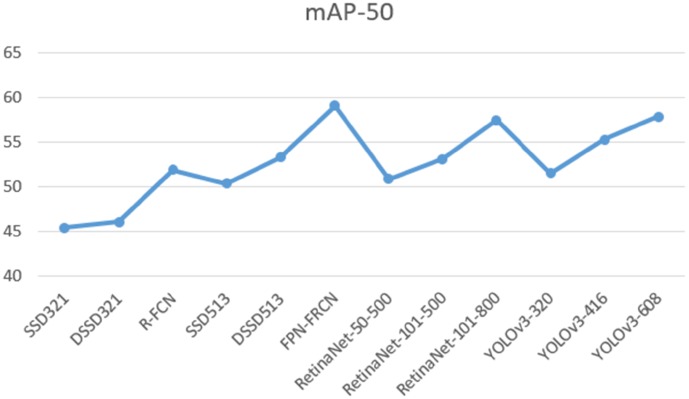
Performance on mAp-50 Yolo-v3 and other deep models on COCO dataset [19].

In identifying cholelithiasis and classifying gallstones on CT images, it needs to record the position, size, shape and type of the gallstones. Statistically, it needs about 25-35s by an experienced doctor to detect gallstones from CT images. If the running time of our deep learning model is too long, its function becomes weak and helpless. It is shown in Figs 1 and 2 the performances of Yolo-v3 and other deep models on COCO dataset [19]. Yolo-v3 and FPN-FRCN can achieve 57.9% and 59.1% in mAP-50, but the running time of FPN-FRCN is 3.4 times of Yolo-v3. It is found that Yolo-v3 model can achieve well performance when the amount of classes is not too big. Since it has about 200 CT images in each time CT examination, Yolo-v3 may use approximately 4 seconds (according to the FPS) to detect gallstones, which is 6-8 times faster than doctors.

We choose here Yolo v3 framework to identify cholelithiasis and classify gallstones on CT images, but the accuracy of identifying cholelithiasis and classifying gallstones on CT images should be improved.

Yolo has only convolutional layers, making it a fully convolutional network (FCN). It has 75 convolutional layers, with skip connections and up sampling layers. No pooling is used, and a convolutional layer with stride 2 is used to down sample the feature maps. This helps in preventing the loss of low-level features often attributed to pooling. Yolo v3 has three anchors, which generates prediction of three bounding boxes per cell.

**Fig 3:**
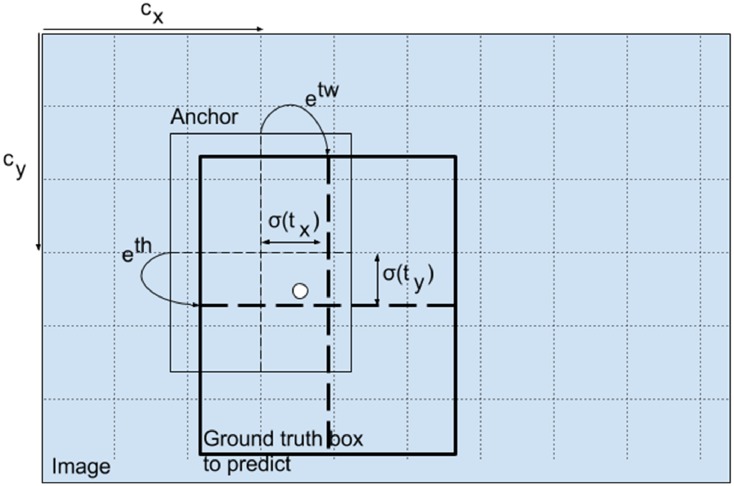
Dimensions of the bounding box.

The dimensions of the bounding box is shown in Fig 3, which are predicted by applying a log-space transform to the output and then multiplying with an anchor. The predicted results are normalised by the height and width of the image. For any predicted *bx* and *by* box, if it contains target in (*bx*, *by*), then the actual width and height of the box is (13 × *bx*, 13 × *by*) on the 13 × 13 feature map. It used sigmoid to calculate the class scores, i.e., the value of class confidences. We improve the class confidences outputs by using specific strategies to improve the accuracy of gallstone detection.

### Gallstone identifying model

Gallstones generally occur in gall and gall nearby the liver, which are obviously different with other organs for neural network to detect. The location information of spine, liver and gall is stable and helpful in recognizing and locating gallstones. We improve our model of identifying gallstones by locating the following five objects.

**Fig 4:**
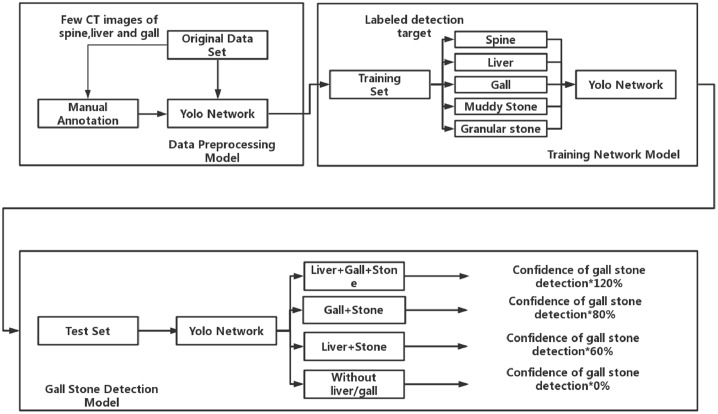
The main structure of our model.

1. **Objects locating**
  - **Spine**: The features of spine can be easily observed and there is spine area in each CT image.
  - **Liver**: Liver is basis of the identification of gallstones, but is not the most important basis.
  - **Gall**: The organ nearby liver has regular forms, which is the place gallstone occurs to, is the most important basis for the identification of gallstone.
  - **Granular gallstones**: They are in white and bright in CT images with irregular shapes.
  - **Muddy gallstones**: They are in gray bulk form on CT images. It is a little darker than the color of the gallbladder, and is difficult to recognize.
2. **Confidence setting policy**
  - Liver and gallbladder can be identified at the same time as stones, and the confidence of stones in the gallbladder can be increased from the original basis until 100%.
  - Only the gallbladder can be identified at the same time as the stone. Meanwhile, the confidence of the stone in the gallbladder can remain unchanged or decrease slightly.
  - When only liver is identified with gallstones, the confidence can be reduced to a certain extent to reduce the possibility that the target is gallstones, but it cannot be zero.
  - Gallstones were identified, but the liver and gallbladder are not, confidence will be set as zero. Such stones are most likely to be misdiagnosed, the strategy that helps reduce the likelihood that non-gallstones will be misjudged.
3. **Dataset**
  We have collected 223846 original abdominal CT images of 1369 patients from year 2011 to 2017, among which approximately 5986 CT images are selected to train our YOLOv3-arch model. Each of the CT images contains spine, liver, gall, granular stone and muddy stone by using data preprocessing model. The 5986 CT images are separated into three groups, in which 4000 CT images are taken as training set, 986 CT images are taken as validation set, 1000 CT images are taken as testing set. This data set contains CT images of granular stones, muddy stones and normal CT images, and no stone containing CT images.
4. **Data pre-processing**. The experimental data set used here is obtained as follows:
  - Collecting 1065 CT images with livers and gallbladders.
  - The 1065 CT images are used to train and test a neural network. After 7,000 iterations, its recognition accuracy achieves 97.3% for the liver, 94.3% for the gallbladder and 95.8% for the average.
  - The trained neural network is used to select 6618 CT images from 223846 CT images.
  - The gallstones and cholelithiasis on 6,618 CT images were labeled, among which 4,986 images were manually obtained for training our YOLOv3-arch model.
5. **Cholelithiasis identification and gallstones classification**
  Different from the general object detection, cholelithiasis and gallstones occur in gall along with liver. We use Yolo to identify the spine, liver, gall and gallstone, and our confidence setting policy to ensure the confidence of objects relates to the relevant things appear or not. In this way, we can improve identification accuracy, especially enhance the accuracy of recognizing negative sample. For instance, the gallstone is detected and the confidence is very high, while liver and gall could not be detected in this CT image, then then confidence of gallstone is set to be 0%. The reason is that gallstone cannot occur separately from liver and gall. In this situation, neural network may occur under-fitting or over-fitting, but if we improve or deduce the training times, it might present a low performance on normal gallstone detection in a gall.
  The main structure of our model is shown in Fig 4. Firstly, we annotate a small amount of CT images which contain spine, liver and gall to train a Yolo network. With the trained neural network, we can pick out CT images as training set. A novel YOLOv3-arch model is trained to identify cholelithiasis and classify gallstones. A coefficient to output the confidence of gallstone is related to the probability of gallstone occurred. If liver, gall, and gallstones are identified at the same time, the confidence of gallstone detection is the final confidence. If only gall or liver is detected with gallstone, we cannot ensure this confidence to be the final confidence, and reduce the probability. If liver and gall are not identified, it is taken as misjudged.
6. **Mean Average Precision (mAP)**.
  Since there are several categories to identify, mAP is a well criterion to evaluate the result of gallstone recognition.

## Results

**Fig 5:**
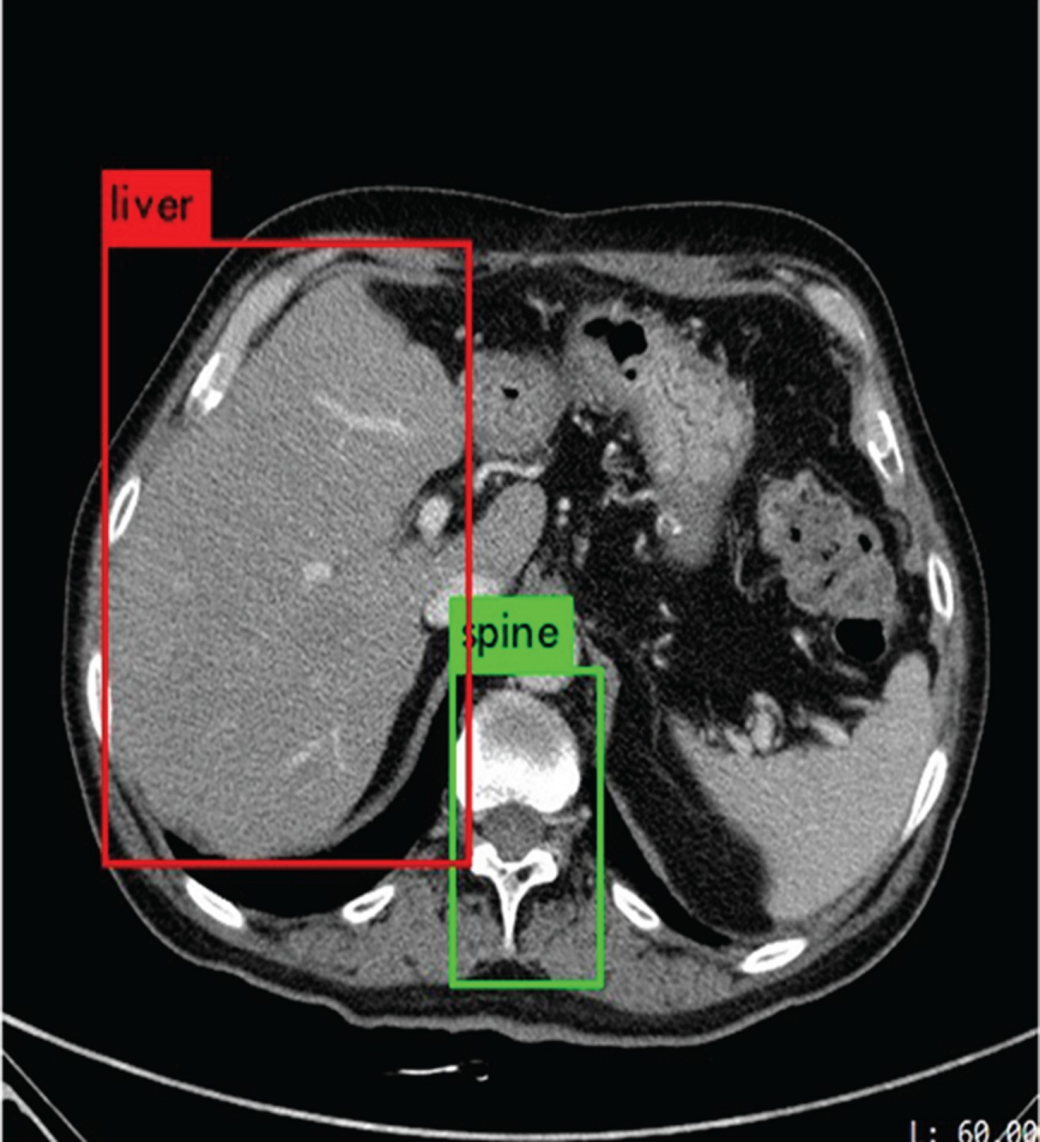
A correctly recognized negative sample.

It is shown in Fig 5 a correctly recognized negative sample. A negative sample is a CT image without gallstones. In the test set, we collect 96 CT images as negative samples. Through the correlation analysis of gallstones location information strategy, we change the confidence level of output and no image output bounding box.

The size of CT images is 512 × 512. A general Yolo v3 neural network misjudges a gray color piece of liver as muddy stone. The confidence of this misjudge is 33%, more than 25%, so it outputs a bounding box (if the confidence more 25%, bounding box will be output). As we can see, there is no presence of the gallbladder. According to the strategy to adjust the final confidence is 33% × 0.6 = 19.8% < 25%, so cancel the output bounding box. The remaining 27 cases misjudged negative samples as positive samples were similar. Confidence is not very high, similar negative samples are between 25% and 40%. It is set the value 0.6 to be a weight to judge present bounding box or not.

We conducted experiments and use cross validation to prove that our YOLOv3-arch model is much more effective than general Yolo v3.

Table 1 shows 10-fold cross-validation of general Yolo v3 model, and conducted a total of 10 experiments on the data set composed of 4986 CT images. The average value of the 10 experiments is recorded. In Table 1, the recognition accuracy of liver and gallbladder is relatively high. The correlation detection is based on the location information of liver and gallbladder, so it is necessary to have a high recognition accuracy of liver and gallbladder. In the identification of gallstones, accuracy of the identifying granular gallstones is relatively high, stable at about 90%, with a verification average of 89.2%. The accuracy of the identifying muddy gallstones is relatively poor, with only 64% at the lowest time and an average of only 72.3%. The average accuracy of stone recognition is 80.75%.

**Table 1:** The precision of general Yolo network in 10-fold cross validation.

Training time	Liver	Gall	Granular stone	Muddy stone	Spine	mAP(all)	mAP(stones)
1	**98**%	93%	90%	76%	96%	90.43%	83.00%
2	97%	93%	88%	72%	96%	89.19%	80.00%
3	96%	92%	90%	66%	96%	88.02%	78.00%
4	97%	97%	88%	64%	96%	88.30%	76.00%
5	**98**%	97%	87%	**84**%	96%	**92.33**%	**85.50**%
6	97%	90%	91%	75%	95%	89.48%	83.00%
7	97%	**98**%	**92**%	72%	94%	90.67%	82.00%
8	96%	95%	89%	65%	94%	87.84%	77.00%
9	**98**%	94%	86%	75%	94%	89.49%	80.50%
10	**98**%	94%	91%	74%	**97**%	91.35%	82.50%
average	97.30%	94.30%	89.2%	72.3%	95.4%	89.71%	80.75%

It is shown in Table 2 the 10-fold cross-validation for our YOLOv3-arch model. It conducts a total of 10 experiments on the data set composed of 4986 CT images, and finally took the average result of 10 experiments. The recognition accuracy of liver and gallbladder is relatively high, with an average of more than 95%. The highest recognition accuracy of granular gallstones is 98%, with an average of 92.7%. It is with the highest identification accuracy of muddy gallstones to be 87%, and with an average of 80.3%. The average detection accuracy of gallstones is 86.50%.

**Table 2:** The precision of the novel gallstones detection model in 10-fold cross validation.

Training time	Liver	Gall	Granular stone	Muddy stone	Spine	mAP(all)	mAP(stones)
1	97%	97%	96%	83%	98%	94.37%	**89.50**%
2	97%	97%	93%	73%	96%	91.15%	83.00%
3	97%	99%	92%	76%	96%	91.78%	84.00%
4	97%	94%	90%	84%	96%	92.40%	87.00%
5	96%	97%	88%	**87%**	93%	92.12%	87.50%
6	98%	95%	93%	81%	94%	92.19%	87.00%
7	97%	97%	97%	81%	96%	93.54%	89.00%
8	96%	94%	**98**%	76%	96%	92.10%	87.00%
9	97%	94%	94%	81%	95%	92.34%	87.50%
10	97%	94%	86%	81%	95%	90.82%	83.50%
average	96.9%	95.8%	92.7%	80.3%	95.50%	92.28%	86.50%

It is obtained from Tables 1 and 2 that our Yolov3-arch model is with average accuracy 92.7% in identifying granular gallstones and average accuracy 80.3% in identifying muddy gallstones. This achieves 3.5% and 8% improvements in identifying granular and muddy gallstones to general Yolo v3 model, respectively. Also, the average cholelithiasis identifying accuracy is improved to 86.50% from 80.75%. Meanwhile, our method can reduces the misdiagnosis rate of negative samples by the object detection model.

YOlOv3-arch model can identify the suspected location of gallstones in the CT images of patients in a very short time. In this experiment, there were 1000 images in the Yolo v3 consensus, 5 targets per image, and the recognition speed was about 8ms per image. For patients with 200 CT images, it only took about 1.5s to identify the location of gallstones, which was about 20-30 times faster than the manual CT images reading time.

## Conclusion

In this paper, we collected more than 223846 CT images with gallstone of 1369 patients from The Third Hospital of Shandong Province, and then a data cleaning method is proposed to automatically select CT images in high quality. After that, we develop a deep learning method, Yolov3-arch neural network, for gallstones recognition. Using the method, the location of gallstones can be automatically marked, as well as the type can be identified. Experimental results show that our method can achieve accuracy 86.5% in recognizing both the type and location of gallstones, which performs better than classical Yolo neural networks. Meanwhile, our method can reduces the misdiagnosis rate of negative samples by the object detection model. According to the time of gallstones’ detection with our model, a patient’s CT images (about 200) may use approximately 4 seconds and it is 3-8 times faster than doctors are. In this way, we can save much time of doctors in searching gallstones.

For further research, the improvement of gallstone detection’s precision and MAP is necessary. Furthermore, the gallstone auto diagnoses should contain the gallstone auto detection and give the diagnostic reports and medical or surgical suggestion, it will request the system could process and output nature language. As well, some spiking neural networks and spiking neural P systems, see e.g. [20–24], can be used as novel tools in gallstone detection.
